# Modular Hyperthermostable Bacterial Endo-β-1,4-Mannanase: Molecular Shape, Flexibility and Temperature-Dependent Conformational Changes

**DOI:** 10.1371/journal.pone.0092996

**Published:** 2014-03-26

**Authors:** Viviam M. da Silva, Francieli Colussi, Mario de Oliveira Neto, Antonio S. K. Braz, Fabio M. Squina, Cristiano L. P. Oliveira, Wanius Garcia

**Affiliations:** 1 Centro de Ciências Naturais e Humanas, Universidade Federal do ABC (UFABC), Santo André, São Paulo, Brazil; 2 Departamento de Física e Biofísica, Instituto de Biociências, Universidade Estadual Paulista, Botucatu, São Paulo, Brazil; 3 Laboratório Nacional de Ciência e Tecnologia do Bioetanol, Centro Nacional de Pesquisa em Energia e Materiais, Campinas, São Paulo, Brazil; 4 Instituto de Física, Universidade de São Paulo, São Paulo, Brazil; Oak Ridge National Laboratory, United States of America

## Abstract

Endo-β-1,4-mannanase from *Thermotoga petrophila* (TpMan) is a hyperthermostable enzyme that catalyzes the hydrolysis of β-1,4-mannoside linkages in various mannan-containing polysaccharides. A recent study reported that TpMan is composed of a GH5 catalytic domain joined by a linker to a carbohydrate-binding domain. However, at this moment, there is no three-dimensional structure determined for TpMan. Little is known about the conformation of the TpMan as well as the role of the length and flexibility of the linker on the spatial arrangement of the constitutive domains. In this study, we report the first structural characterization of the entire TpMan by small-angle X-ray scattering combined with the three-dimensional structures of the individual domains in order to shed light on the low-resolution model, overall dimensions, and flexibility of this modular enzyme at different temperatures. The results are consistent with a linker with a compact structure and that occupies a small volume with respect to its large number of amino acids. Furthermore, at 20°C the results are consistent with a model where TpMan is a molecule composed of three distinct domains and that presents some level of molecular flexibility in solution. Even though the full enzyme has some degree of molecular flexibility, there might be a preferable conformation, which could be described by the rigid-body modeling procedure. Finally, the results indicate that TpMan undergoes a temperature-driven transition between conformational states without a significant disruption of its secondary structure. Our results suggest that the linker can optimize the geometry between the other two domains with respect to the substrate at high temperatures. These studies should provide a useful basis for future biophysical studies of entire TpMan.

## Introduction

Lignocellulosic biomass is the major renewable carbon source in nature with several applications and consists of cellulose, hemicellulose and lignin with an approximate representation of 2∶1:1 [1]–[3]. Cellulose consists of linear polysaccharides of β-1,4-linked D-glucose residues, whereas lignin is a phenolic macromolecule. Hemicelluloses are linear or branched heteropolysaccharides derived from sugars such as D-xylose, D-galactose, D-mannose, D-glucose, and L-arabinose [4]. They are classified according to the main sugar unit as xylans, galactans and mannans, and most of the main-chain sugars on hemicellulose polymer are linked together by β-1,4-glycosidic bonds [4]–[6]. Xylans comprise the major hemicellulose component present in hardwoods, whereas mannans are more prominent in softwoods [5], [7]. Mannans are classified into two principal groups depending on whether the β-1,4-linked backbone contains only D-mannose (mannans) or a combination of D-mannose and D-glucose residues (glucomannans). Linear mannan or glucomannan chains containing more than 5% D-galactose are called galactomannans and galactoglucomannans, respectively [7].

The biodegradation of cellulose and hemicellulose polymers involves the concerted action of a variety of hydrolytic enzymes. Cellulases and xylanases have been extensively studied due to their several industrial applications principally relating to biorefineries, however, mannan-degrading enzymes have enjoyed less attention. The major enzymes involved in the biodegradation of mannan polysaccharides are β-mannanase (EC 3.2.178), β-mannosidase (EC 3.2.1.25), and β-glucosidase (EC 3.2.1.21) [7], [8]. The enzyme β-mannanase is responsible for the cleavage of β-1,4-linked internal linkages of the mannan polymer to produce new chain ends, whereas β-mannosidase cleaves β-1,4-linked mannosides releasing mannose from the nonreducing end of mannans and mannooligosaccharides [7], [8]. The enzyme β-glucosidase hydrolyzes 1,4-β-D-glucopyranose at the nonreducing end of the oligosaccharides released from glucomannan and galactoglucomannans by β-mannanase. Additional enzymes such as acetyl mannan esterase (EC 3.1.1.6) and α-galactosidase (EC 3.2.1.22) are required to remove side groups that might be attached at several points on the mannan polymer, creating more sites for subsequent enzyme hydrolysis [7], [8]. The biodegradation of mannan represents a key step for various industrial applications including delignification of kraft pulps, food processing and production of second-generation biofuels [6]–[11].

*Thermotoga petrophila* (*T. petrophila*) strain RKU-1 (T) is a hyperthermophilic bacterium isolated from the Kubiki oil reservoir in Niigata (Japan) [12]. The temperature range for growth is 47–88°C with an optimum at 80°C. The pH range for growth is 5–9 with the optimum at pH 7. This bacterium produces a repertoire of hyperthermostable enzymes of great potential for industrial applications, including cellulases, arabinofuranosidases, arabinanases and mannanases, and has proved to be a suitable source of enzymes for biotechnological applications and protein engineering of glycoside hydrolases [13]–[15]. For various industrial applications, it is essential that mannanase possesses high activity and thermostability [11].

The hyperthermostable bacterial endo-β-1,4-mannanase (EC 3.2.1.78) from *T. petrophila* (TpMan) is an enzyme composed of 667 amino acid residues (Gene Bank: ABQ47550.1). The first 20 amino acid residues are a signal peptide, which are removed after protein exportation. TpMan enzyme presents optimal activity at the temperature range of 81–93°C [13]. Both TpMan and its catalytic domain present maximal activity at the acidic pH range of 4.5–6.5. The truncated catalytic domain had optimal activity at a lower temperature range when compared to the TpMan enzyme [13]. A recent study reported that TpMan consists of a GH5 catalytic domain (TpManGH5, 373 amino acid residues) and a carbohydrate-binding domain (TpManCBM27, 172 amino acid residues) connected through a linker (102 amino acid residues) [13]. Furthermore, the same study presented the crystal structure of GH5 catalytic domain, however, at this moment, there is no three-dimensional structure available for entire TpMan.

In the present study, the bacterial TpMan was analyzed using biophysical techniques as well as with bioinformatics tools. Here, we report the first structural characterization of hyperthermostable endo-β-1,4-mannanase from the bacterium *T. petrophila* by small-angle X-ray scattering in order to shed light on the low-resolution molecular envelope, overall dimensions, and flexibility of this modular enzyme at different temperatures.

## Results

### Temperature Profile of TpMan Enzymatic Activity

**Figure 1:**
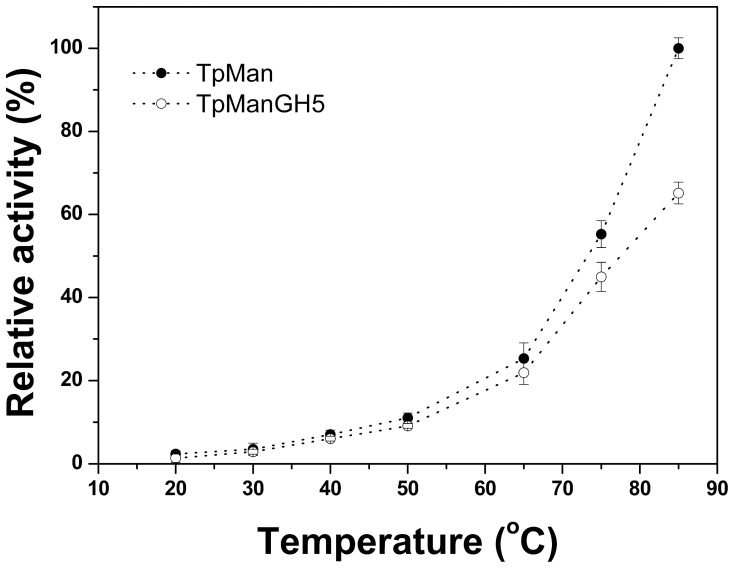
Enzymatic activity. Effect of temperature on the enzymatic activity of TpMan and TpManGH5.

The activity of an enzyme is dependent on pH and temperature, conditions that invoke changes in enzyme folding, conformation, and protonation. As a first step of our studies, the mannan endo-1,4-β-mannosidase activity of TpMan and TpManGH5 were studied as a function of temperature using locust bean gum (galactomannan) as substrate (Fig. 1). Our results showed that, under the conditions of our study (at pH 6), the enzymatic activity of TpMan increases as temperature increases. A similar behavior was also observed in the case of TpManGH5. However, TpManGH5 showed a significant decline in the enzymatic activity at 85°C when compared to TpMan (Fig. 1). Thus, our results indicate that TpMan is more thermotolerant than the truncated catalytic domain. The results presented here are consistent with the results published recently for TpMan [13].

### Dynamic Light Scattering (DLS)

**Figure 2:**
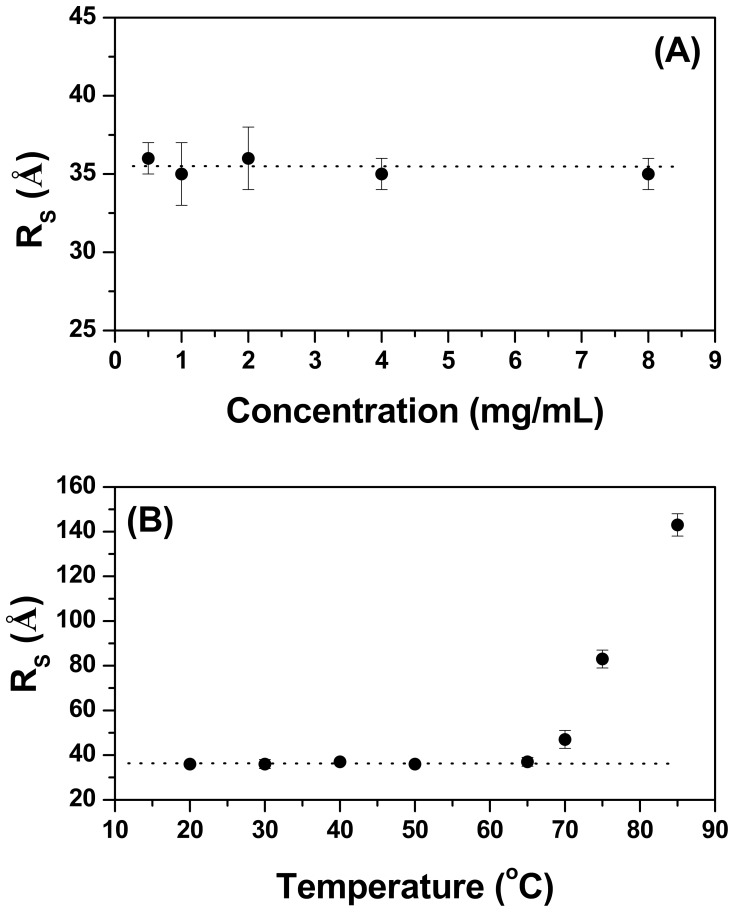
Characterization of TpMan by DLS at pH 6. (**A**) The hydrodynamic radius (*R_S_*) of TpMan (at 20°C) as a function of the protein concentration. (**B**) The hydrodynamic radius (*R_S_*) of TpMan (at 8 mg/mL) as a function of temperature.

**Figure 3:**
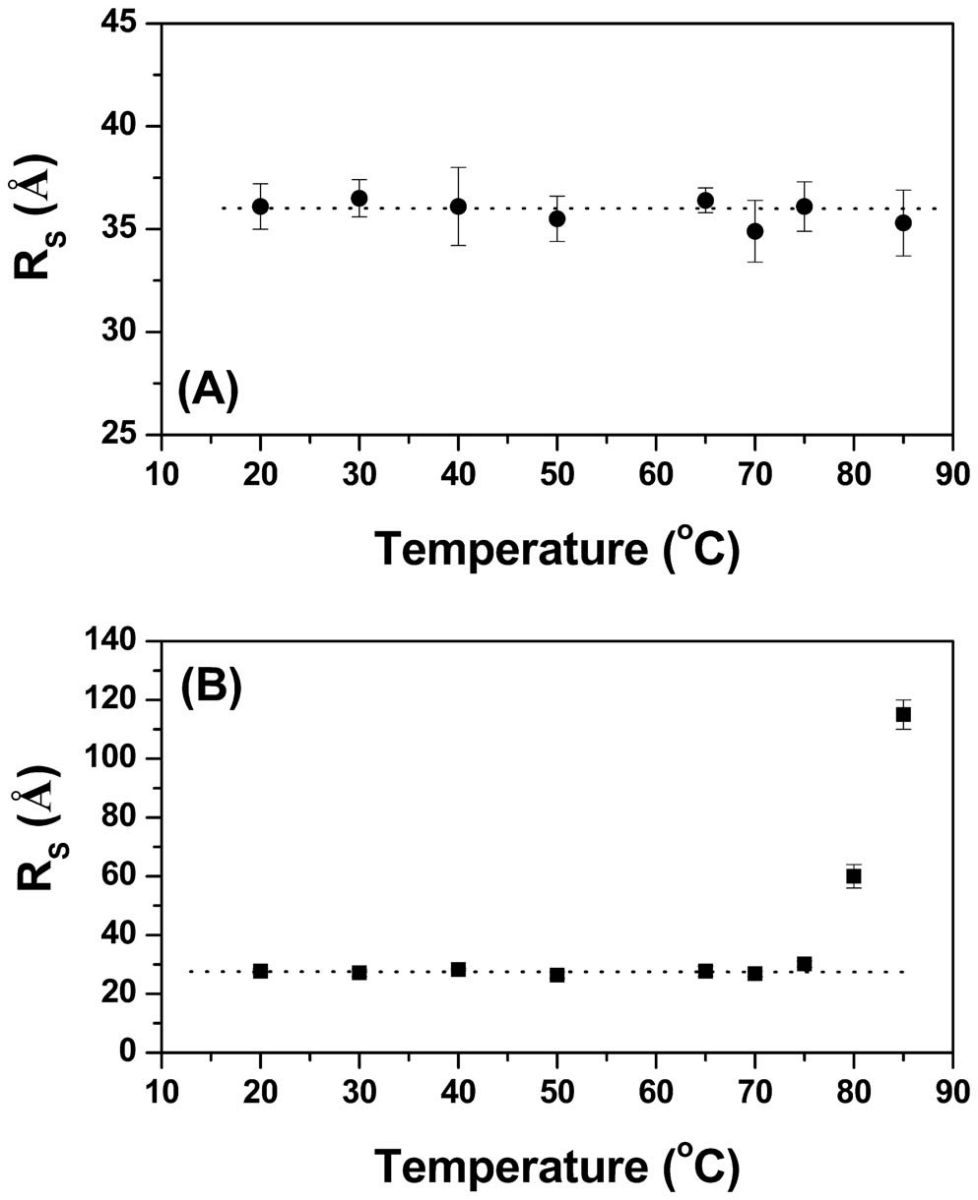
Characterization of TpMan and TpManGH5 by DLS at pH 6. (**A**) The hydrodynamic radius (*R_S_*) of TpMan (at 0.5 mg/mL) as a function of temperature. (**B**) The hydrodynamic radius (*R_S_*) of TpManGH5 (at 0.5 mg/mL) as a function of temperature.

Dynamic light scattering (DLS) provides a fast and convenient way to monitor the size of proteins in solution and also investigate whether the enzyme aggregates at high temperatures and/or at high protein concentrations [16]. DLS size distribution profiles for TpMan and TpManGH5 are available in supporting information (Figs. S1 and S2). As can be seen, the hydrodynamic radii (*R_S_*) of TpMan were practically independent of protein concentration over the range 0.5 to 8 mg/mL at 20°C and pH 6 (Fig. 2A). Furthermore, the *R_S_* of TpMan exhibited minimal temperature dependence over the range 20 to 85°C at pH 6 and 0.5 mg/mL (Fig. 3A). After incubation at 85°C (at 0.5 mg/mL) there was no evidence of enzyme precipitation and the solution remained transparent. The average value of *R_S_* determined for TpMan by the DLS method was 36±2 Å, which corresponds to a molecular mass of 68±6 kDa, consistent with the expected molecular mass of 73 kDa. However, when TpMan at pH 6 and 8 mg/mL was incubated at temperature values above 65°C the *R_S_* increased significantly, suggesting a tendency to form amorphous aggregates (Fig. 2B) [16]. After incubation at 85°C (at 8 mg/mL) the initially clear TpMan solution was very turbid and precipitate had formed.

In the case of TpManGH5, the *R_s_* were practically independent of temperature over the range 20 to 75°C at pH 6 and 0.5 mg/mL (Fig. 3B). The average *R_S_* determined for TpManGH5 by the DLS method was 28±2 Å, which corresponds to a molecular mass of 38±5 kDa, entirely consistent with the expected molecular mass of 42 kDa. However, when TpManGH5 at pH 6 and 0.5 mg/mL was incubated at temperature values above 75°C the *R_S_* increased significantly, suggesting a tendency to form amorphous aggregates (Fig. 3B). Again, after incubation at 85°C (at 0.5 mg/mL) the initially clear TpManGH5 solution was very turbid and precipitate had formed.

### Small-Angle X-ray Scattering Data Analysis and Low Resolution Modeling

**Figure 4:**
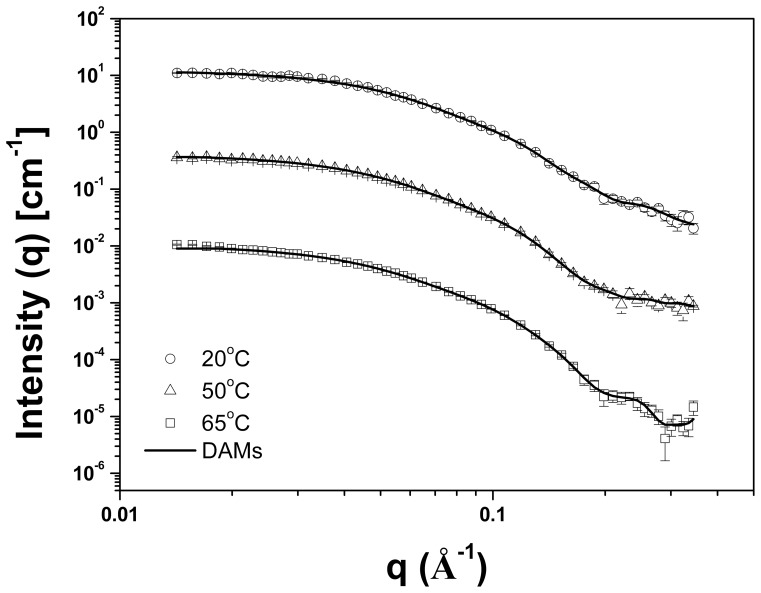
SAXS data collected for TpMan at pH6 and different temperatures. Experimental SAXS curves of the TpMan at 20°C (open black circles with errors bars), 50°C (open black triangles with errors bars) and 65°C (open black squares with errors bars) superimposed on the computed scattering curves based on the restored low-resolution models (DAMs, solid black lines). The curves have been offset for clarity.

**Figure 5:**
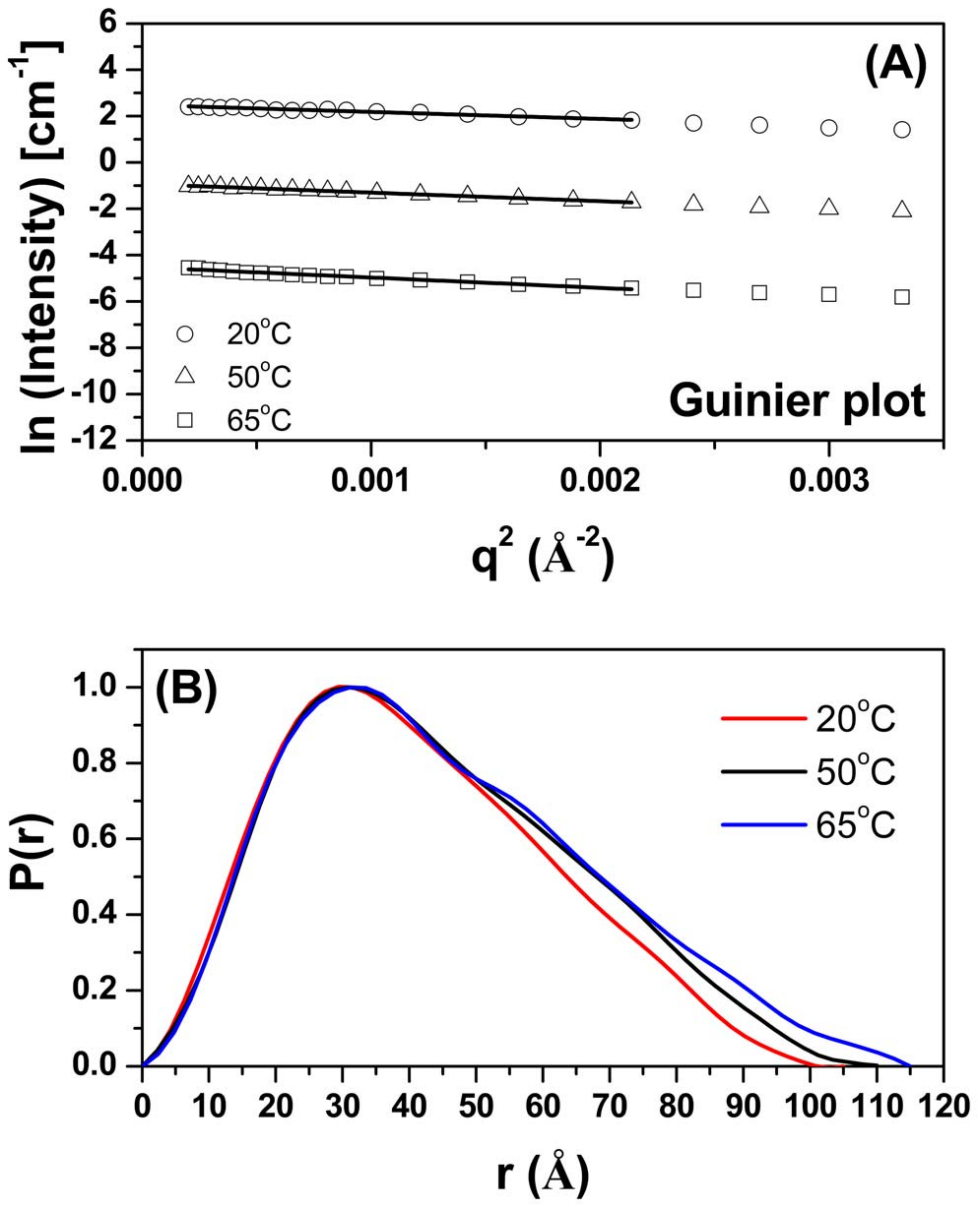
Guinier plots and experimental distance distribution functions. (**A**) Guinier plots (ln *I* versus *q^2^*) for TpMan at 20°C (open black circles), 50°C (open black triangles) and 65°C (open black squares). The curves have been offset for clarity. (**B**) Experimental *P*(r) of TpMan at 20°C (red solid line), 50°C (black solid line) and 65°C (blue solid line). The curves have been scaled to a maximum height of 1.0 for ready comparison.

To obtain more detailed information about the tertiary structure of TpMan and its molecular shape, we submitted TpMan to small-angle X-ray scattering (SAXS) analysis as a function of temperature. The X-ray scattering curves obtained at 8 mg/mL and three different temperatures (T = 20, 50 and 65°C) for TpMan at pH 6 are shown in Fig. 4. Scattering curves obtained at 80°C and higher concentration showed nonnegligible interference effects, and they were not used for analyzes (data not shown). The Guinier plots of the data exhibited a linear behavior indicating excellent monodispersity of TpMan and that the particles are free of significant aggregation (Fig. 5A). Furthermore, the variation among the radii of gyration values for different protein concentrations and same temperature were insignificants (Table 1). The radii of gyration (*R_g_*) evaluated with the Guinier approximation at 20, 50 and 65°C were 32±1 Å, 34±1 Å and 36±1 Å, respectively. The pair distance distribution functions, *P*(r), evaluated by the indirect Fourier transform with GNOM package [17] are shown in Fig. 5B. The profiles obtained at 20, 50 and 65°C have very similar shapes and trail off to a maximum dimension (*D_max_*) of 105±5 Å, 110±5 Å and 115±5 Å, respectively. The *R_g_* values determined for TpMan at 20, 50 and 65°C using the GNOM package were 32.50±0.14 Å, 34.87±0.15 Å and 35.98±0.19 Å, respectively.

**Table 1:** Radius of gyration (*R_g_*) calculated with Guinier approximation at different protein concentrations.

Conc. (mg/mL)	*R_g_* (Å) at 20°C	*R_g_* (Å) at 50°C	*R_g_* (Å) at 65°C
2	32±1	33±1	36±1
4	33±1	34±1	35±1
8	32±1	34±1	36±1

**Figure 6:**
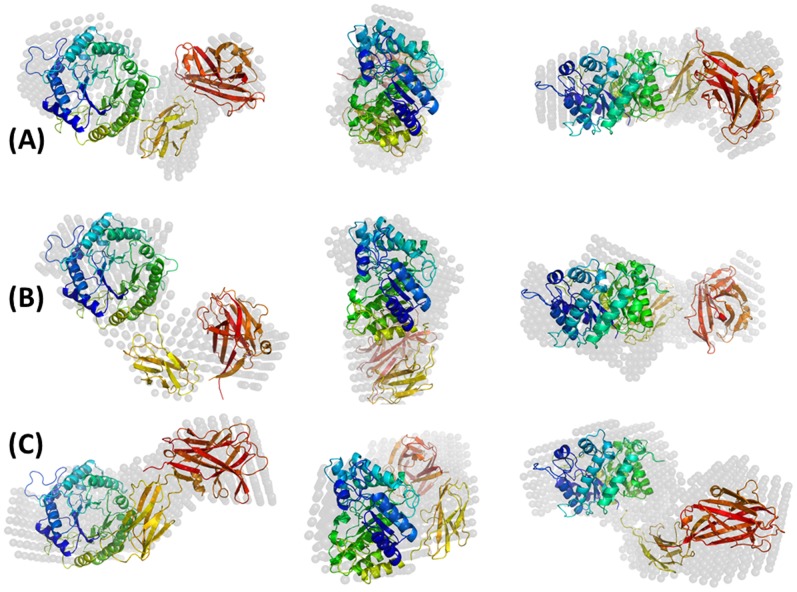
Low-resolution models. (**A**) Molecular envelope of TpMan in solution at 20°C obtained by DAMMIN package (dummy atoms). The center and right structures were rotated y axis-90°and × axis-90° in relation to the left structures. (**B**) Molecular envelope of TpMan in solution at 50°C obtained by DAMMIN package (dummy atoms). The center and right structures were rotated y axis-90°and × axis-90° in relation to the left structures. (**C**) Molecular envelope of TpMan in solution at 65°C obtained by DAMMIN package (dummy atoms). The center and right structures were rotated y axis-90°and × axis-90° in relation to the left structures.

The low-resolution molecular shape of TpMan was determined from X-ray scattering data with DAMMIN package [18] to visualize the shape of the enzyme in solution at different temperatures. The resulting consensus envelopes for the temperature values studied are shown in Fig. 6. In each case, ten independent *ab initio* simulations were performed without imposing any symmetry restrictions, and the models described very well the experimental curves. The results, in each case, were compared with each other by the use of the DAMAVER procedure [19] and the most representative model for the whole set is shown in Fig. 6. The reconstructed low-resolution model of TpMan at 20°C and pH 6, restored at 18 Å resolution, showed an elongated L-shaped molecule, with a *D_max_* of approximately 105 Å. However, the reconstructed low-resolution model of TpMan at 65°C and pH 6, restored at 18 Å resolution, showed a more elongated shape roughly twice as long as they are wide, with a *D_max_* of approximately 115 Å. The atomic coordinates of the low-resolution models of TpMan are available by request to the corresponding author of this article. A summary of the main SAXS results described in this study for TpMan is given in Table 2.

**Table 2:** General SAXS results from TpMan.

Parameters/samples	20°C			
	Experimental	DAM	RBM	EOM
***R_g_*** ** (Å)**	32±1 (Guinier)32.50±0.14 (GNOM)	32.97	32.30	31.14
***D_max_*** ** (Å)**	105±5	103.90	104.70	102.70
**Resolution (Å)**	18	–	–	–
**χ/χ^2^**	–	1.6/2.6	1.8/3.2	1.9/3.6
	**50°C**			
	**Experimental**	**DAM**	**RBM**	**EOM**
***R_g_*** ** (Å)**	34±1 (Guinier)34.87±0.15 (GNOM)	35.26	33.99	33.22
***D_max_*** ** (Å)**	110±5	110.60	111.20	105.17
**Resolution (Å)**	18	–	–	–
**χ/χ^2^**	–	1.1/1.2	1.3/1.7	2.4/5.8
	**65°C**			
	**Experimental**	**DAM**	**RBM**	**EOM**
***R_g_*** ** (Å)**	36±1 (Guinier)35.98±0.19 (GNOM)	36.41	34.95	34.31
***D_max_*** ** (Å)**	115±5	116.20	118.20	108.63
**Resolution (Å)**	18	–	–	–
**χ/χ^2^**	–	1.3/1.7	1.5/2.3	2.1/4.4

### Kratky Plot and Flexibility

**Figure 7:**
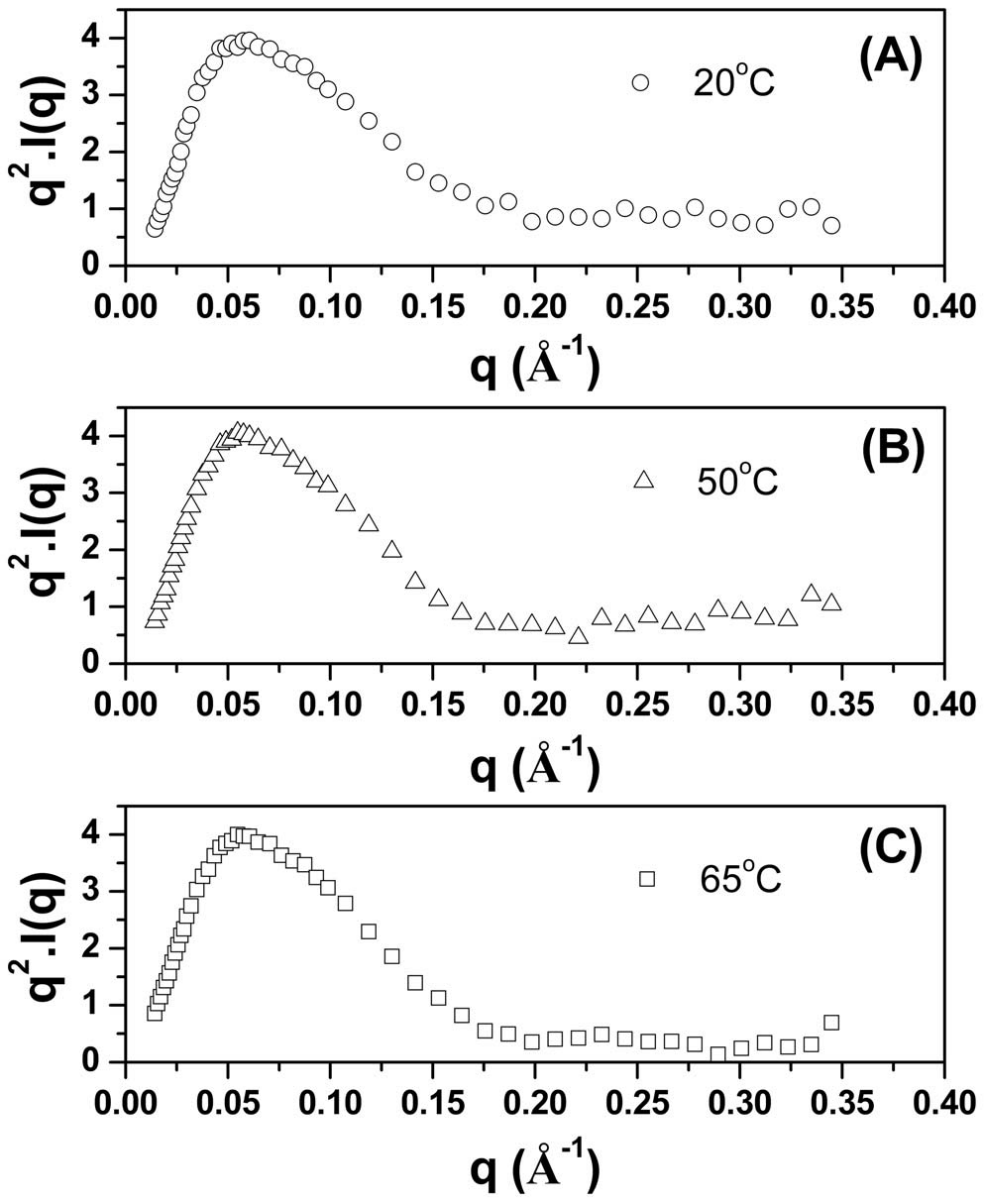
Kratky plots. (**A**) Kratky plot of SAXS data for TpMan at 20°C transformed as *q* ^2^.*I*(*q*) versus *q*. (**B**) Kratky plot of SAXS data for TpMan at 50°C transformed as *q* ^2^.*I*(*q*) versus *q*. (**C**) Kratky plot of SAXS data for TpMan at 65°C transformed as *q* ^2^.*I*(*q*) versus *q*.

Information about the global compactness of TpMan can be obtained from the SAXS data by the use of the so called Kratky plot (*q* ^2^.I vs. *q*) [20], [21]. As can be seen in Fig. 7A, the curve for TpMan at 20°C (at pH 6) shows a well-defined maximum (*q* <0.15 Å^−1^), however, it does not decay close to zero at higher *q* values, presenting a slight elevated baseline. This result suggests that TpMan may exhibit some degree of flexibility at 20°C and pH 6, probably owing to the inherent flexibility between domains. At 50°C and pH 6, the curve shows also a well-defined maximum and presents a subtle decrease of the baseline when compared to the same enzyme at 20°C (Fig. 7B). Interestingly, at 65°C and pH 6 (Fig. 7C) the curve for TpMan decay close to zero at higher *q* values, suggesting that the molecule is less flexible in solution at 65°C when compared to the same enzyme at 20°C.

### Molecular Modeling

**Figure 8:**
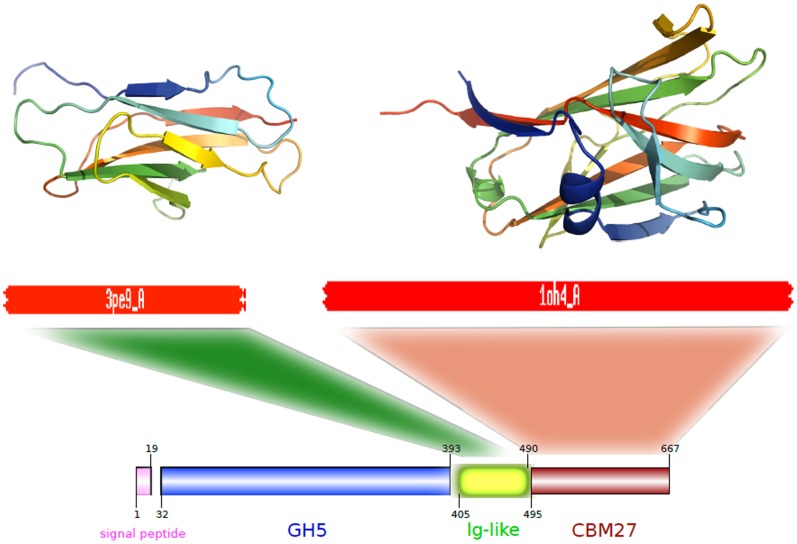
Threading methods and molecular modeling. The templates obtained by threading methods are carbohydrases from thermophilic bacteria. The central domain (TpManIg-like) was directly modeled from to X1 domain from CbhA [23]. TpManCBM27 was directly modeled from to CBM27 from TmMan [22].

A recent study presents the crystal structure of GH5 catalytic domain [13], however, at this moment, there is no three-dimensional structure determined by experimental methods available for entire TpMan. In order to permit the modeling of the entire structure, the three-dimensional structure of the last 274 amino acids residues from TpMan was modeled using bioinformatics tools. As can be seen from Fig. 8 and Table 3, both the threading methods used (HHPRED and PHYRE2) indicated two distinct domains. The C-terminal domain (TpManCBM27) presents high sequence identity and confidence, in both servers, when compared with CBM27 from *Thermotoga maritima* endo-β-1,4-mannanase (TmMan) (Table 3). Therefore, TpManCBM27 was directly modeled (Fig. 8) from its 88% sequence identity to CBM27 from TmMan whose three-dimensional structure was solved by X-ray crystallography [22]. However, the central domain (linker) presents low sequence identity, in both servers, when compared with X1 domain from *Clostridium thermocellum* cellobiohydrolase A (CbhA) [23]. Despite low values of sequence identity obtained in both servers, it is obvious the high level of confidence of the selected template (Table 3). Thus, the central domain was modeled from its 14% sequence identity and 97% confidence to X1 domain from CbhA (Fig. 8). The molecular model obtained for the central domain is structurally very similar to immunoglobulin-like β-sandwich (Ig-like) domain and it will be adopted the nomenclature TpManIg-like for this domain.

**Table 3:** Templates selection by HHPRED and PHYRE2 for central and C-terminal domains.

Templates selection by HHPRED and PHYRE2 for central and C-terminal domains								
Central domain								
**CATH**	**2.60.40**	**Topology:** Immunoglobulin-like						
**SCOP**	**b.1.2.1**	**Fold:** Immunoglobulin-like beta-sandwich						
		**Superfamily:** Fibronectin type III						
**PDBs**	3TP4, 2X2Y, 2BVY, 2BVT		HHPRED				PHYRE2	
**organism**		**name**	**score**	**Ident %**	**Prob**	**E-value**	**conf**	**Ident %**
*Cellulomonas fimi*		MAN26A	111.84	18	99.07	2.8e^1^°	98.7	18
**PDBs**	3PE9, 3PDD, 3PDG		HHPRED				PHYRE2	
**organism**		**name**	**score**	**Ident %**	**Prob**	**E-value**	**conf**	**Ident %**
*Clostridium thermocellum*		X1-module	45.99	14	96.90	0.01	95.9	13
**C-terminal domain**								
**CATH**	**2.60.120.260**	**Topology:** Jelly rolls						
**SCOP**	**b.18.1.18**	**Fold:** Galactose-binding domain-like						
		**Superfamily:** Galactose-binding domain-like						
		**Family:** Family 27 carbohydrate binding module, CBM27						
**PDBs**	1OH4, 1OF3, 1OF4		HHPRED				PHYRE2	
	**organism**	**name**	**score**	**Ident %**	**Prob**	**E-value**	**conf**	**Ident %**
*Thermotoga maritima*		CMB27	376.26	88	100	3e^−54^	100	89
**PDBs**	1PMH, 1PMJ		HHPRED				PHYRE2	
	**organism**	**name**	**score**	**Ident %**	**Prob**	**E-value**	**conf**	**Ident %**
*Caldicellulosiruptor saccharolyticus*		CMB27	300.52	20	100	3e^−54^	100	24

The obtained templates are carbohydrases from thermophilic bacteria.

### Far-UV Circular Dichroism (CD) Spectroscopy

**Figure 9:**
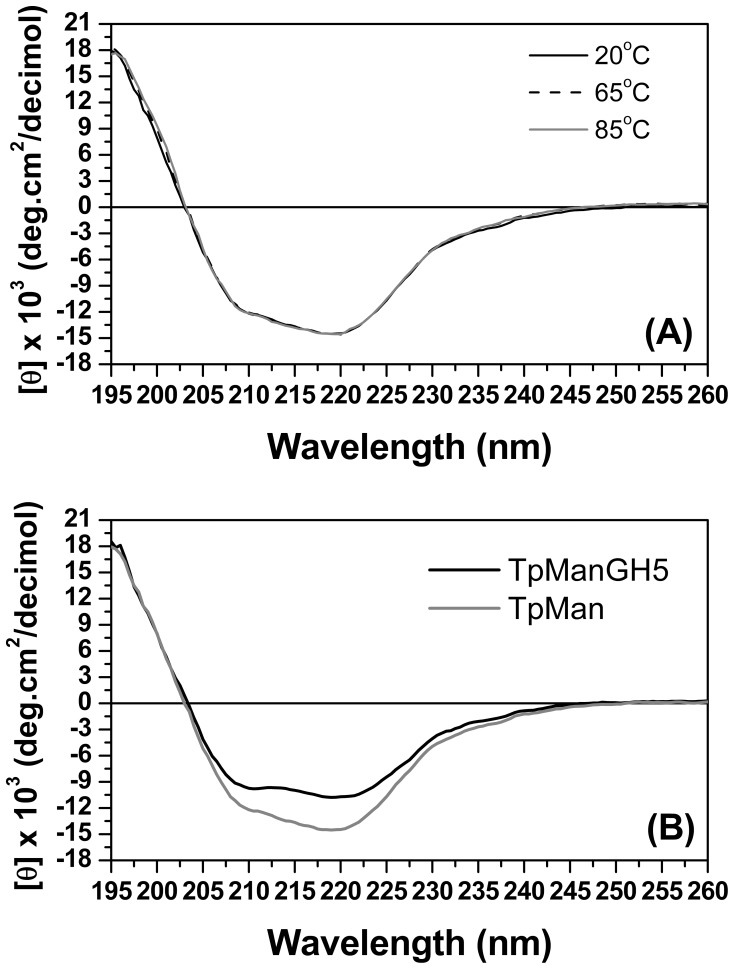
Circular dichroism spectroscopy. (**A**) CD spectrum collected for recombinant TpMan at 20°C (black solid line), 50°C (black dash line) and 85°C (gray solid line). (**B**) CD spectrum of TpManGH5 (black solid line) and TpMan (gray solid line) at pH 6 and 20°C.

Far-UV CD spectroscopy was used to probe the secondary structure of the expressed TpMan and TpManGH5 products to assess their folding in terms of secondary structure and to draw comparisons (Fig. 9). The CD spectrum of TpManGH5 collected at 20°C (pH 6) is characterized by a positive band at 195±1 nm and two negative bands at 210±1 nm and 220±1 nm, together with a negative to positive crossover at 203±1 nm (Fig. 9B). These bands are typically found in structures with a large content of α-helical secondary structure. This is different to that observed for the TpMan CD spectrum, which does not show two minima well-defined. The CD spectra for TpMan collected at pH 6 and three temperature values is shown in Fig. 9A. The data indicate that the secondary structure of the enzyme does not change significantly in response to increasing the temperature from 20 to 85°C.

The percentages of α-helix, β-strand, and coils (turns and irregular structures) were obtained by deconvolution of the experimental spectra using four independent methods and expressed as a mean and standard deviation (see the Materials and Methods). In the case of TpManGH5, the deconvolution of the spectrum yielded the following average values for the secondary structure (Table 4): 42±4% α-helix, 16±2% β-sheet, and 42±5% coil (turn plus random). The corresponding results obtained for TpMan were 21±4% α-helix, 28±3% β-sheet, and 51±5% turns/irregulars structures (Table 4), indicating an increase in β-sheet and coil content and a decrease in α-helix content in the entire enzyme.

**Table 4:** CD deconvolution and secondary-structure prediction.

CD deconvolution*			
**domains**	**α-helix (%)**	**β-sheet (%)**	**coil (%)**
**TpManGH5**	42±4	16±2	42±5
**TpMan**	21±4	28±3	51±5
**Three-dimentional structure** **			
**domains**	**α-helix (%)**	**β-sheet (%)**	**coil (%)**
**TpManGH5**	41	12	47
**TpManIg-like**	0	40	60
**TpManCBM27**	6	60	34
**TpMan**	25	29	46
**Secondary-structure prediction** ***			
**domains**	**α-helix (%)**	**β-sheet (%)**	**coil (%)**
**TpManGH5**	31–40	11–17	45–57
**TpManIg-like**	0–9	42–51	43–57
**TpManCBM27**	0–11	36–60	34–65
**TpMan**	18–26	23–32	39–59

*The percentages of α-helix, β-strand, and coils were obtained by deconvolution of the experimental spectra using four independent methods and expressed as a mean and standard deviation.

**The secondary structures were obtained from the three-dimensional structures of TpManGH5, TpManIg-like and TpManCBM27.

***The computational methods BALDIG, PHD, PSIPRED, and PORTER are compared. The maximum and minimum values obtained for each type of secondary structure are given.

### Computational Methods for Secondary-Structure Prediction

The results of the secondary-structure prediction for the complete TpMan sequence (Gene Bank: ABQ47550.1) and its individual domains are shown in Table 4. The results indicate that overall TpMan is composed of roughly equal percentages of regular secondary structure (α-helices and β-sheets) and random coil (turns and irregular structures). The regular secondary structure of the TpManGH5 is dominated by α-helix. However, the regular secondary structure of the TpManCBM27 and TpManIg-like are dominated by β-sheet.

### Ensemble Optimization Method (EOM) Modeling

**Figure 10:**
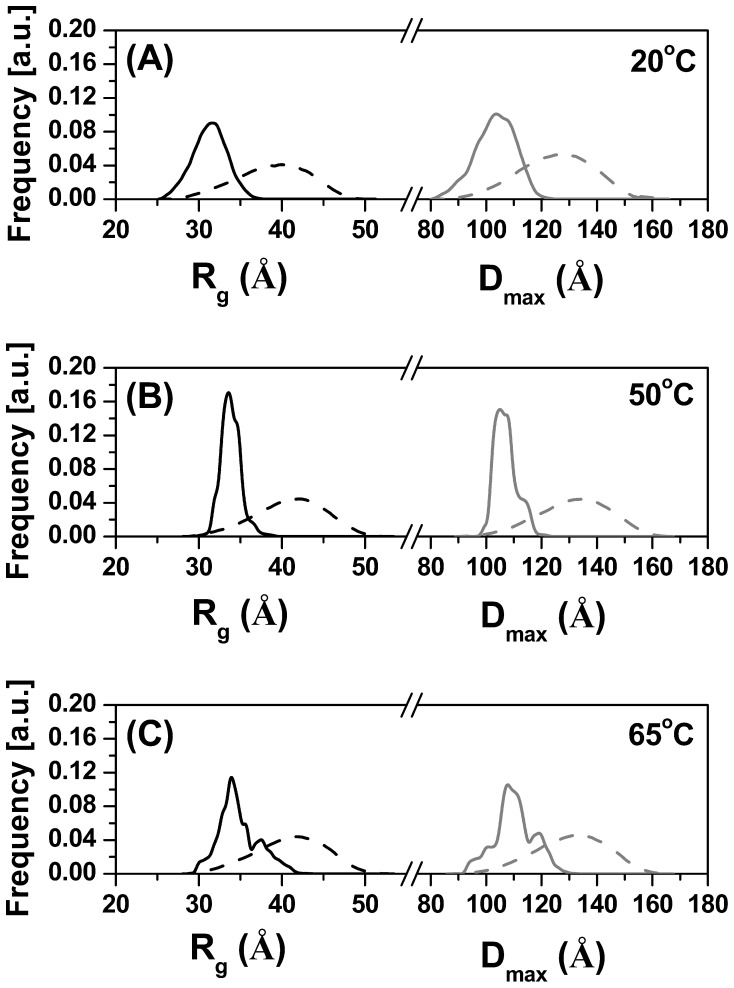
EOM modeling. (**A**) The dash lines are the distributions (*R_g_* and *D_max_*) produced by the software from the SAXS data collected at 20°C, whereas the solid lines are the final distributions of structural parameters from the models selected by the ensemble optimization method [24]. (**B**) The dash lines are the distributions (*R_g_* and *D_max_*) produced by the software from the SAXS data collected at 50°C, whereas the solid lines are the final distributions of structural parameters from the models selected by the EOM [24]. (**C**) The dash lines are the distributions (*R_g_* and *D_max_*) produced by the software from the SAXS data collected at 65°C, whereas the solid lines are the final distributions of structural parameters from the models selected by the EOM.

**Figure 11:**
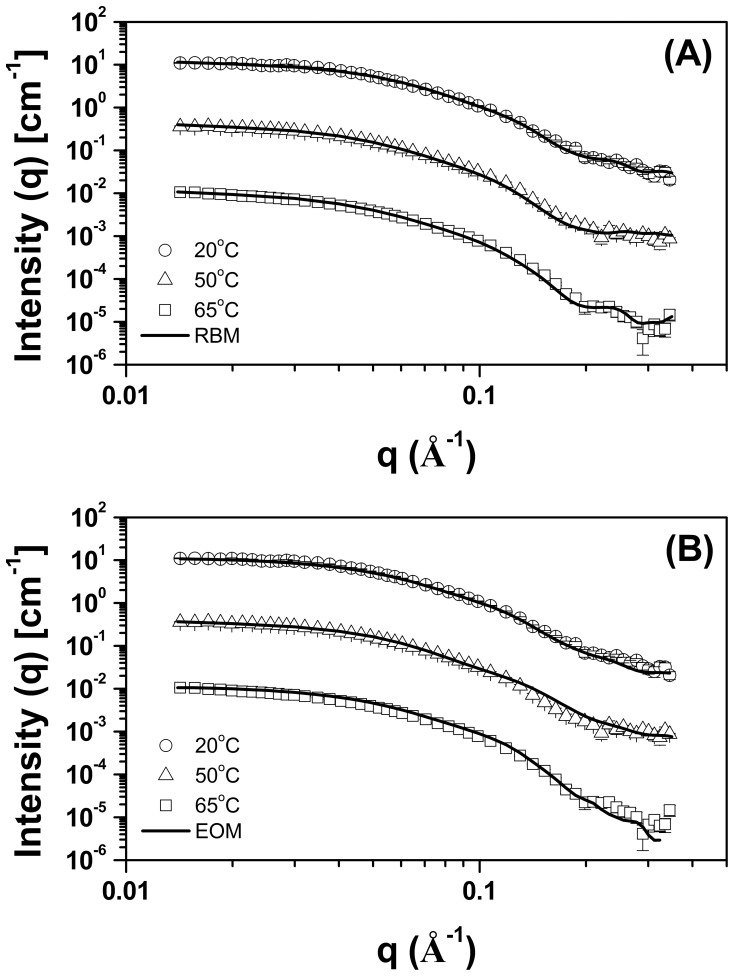
RBM and EOM fits. (**A**) Experimental SAXS curves of the TpMan at 20°C (open black circles with errors bars), 50°C (open black triangles with errors bars) and 65°C (open black squares with errors bars) superimposed on the computed scattering curves based on the rigid-body models (RBMs, solid black lines). (**B**) Fits (solid black lines) of the best profiles determined by EOM modeling to the SAXS data collected at pH 6.0 and 20°C (open black circles with errors bars), 50°C (open black triangles with errors bars) and 65°C (open black squares with errors bars).

**Figure 12:**
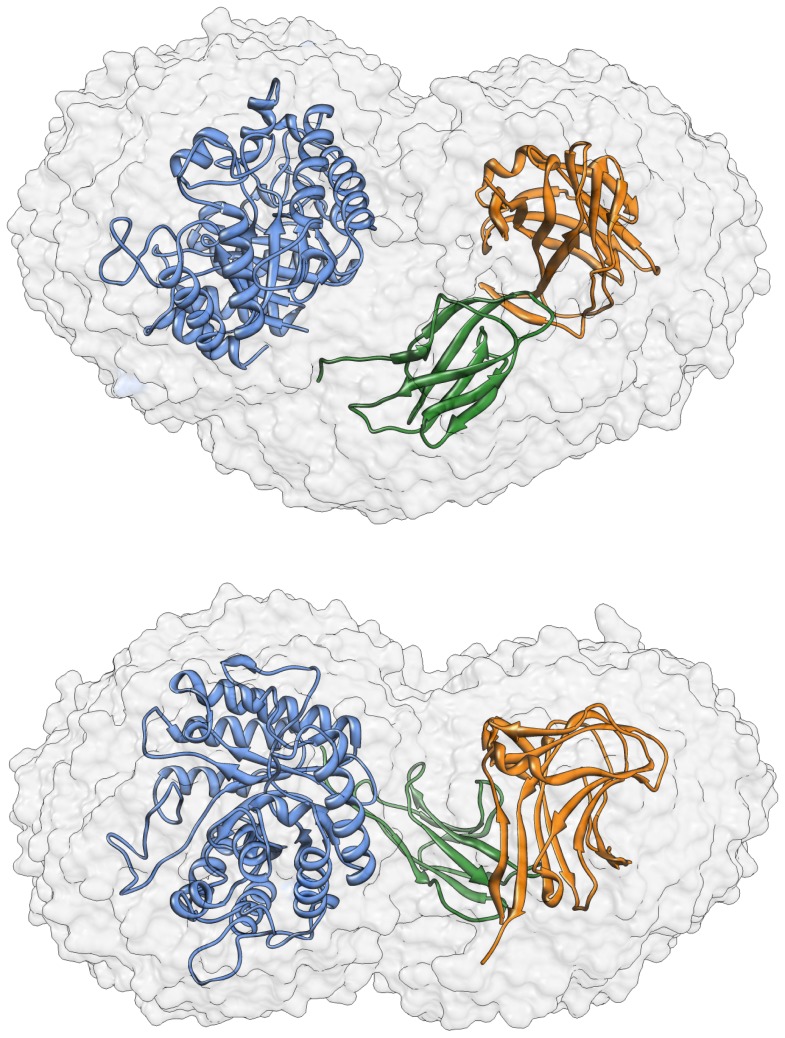
EOM model. Selected model (cartoon) having the highest frequency in the optimized population superposed to the cloud of models. The structure shown is rotated 90°about the long axis of the structure to produce the two views.

To understand better the flexibility information suggests by the Kratky plot at different temperatures, ensemble optimization modeling (EOM) was applied [24]. The *R_g_* and *D_max_* distributions of the structures generated for the initial pool and the distributions of the structures selected during the ensemble optimization are shown for 20, 50 and 65°C in Fig. 10. The selected populations of structures became narrower when the temperature was raised from 20 to 65°C, suggesting a decrease in the molecular flexibility with the increase in temperature. At 20°C the *R_g_* and *D_max_* of the structures selected during the ensemble optimization are centered around 31 Å and 103 Å, respectively (Table 2). At 50°C the *R_g_* and *D_max_* of the structures selected are centered around 33 Å and 105 Å, respectively. Finally, at 65°C the *R_g_* and *D_max_* of the structures selected are centered around 34 Å and 109 Å, respectively. The EOM results indicate that TpMan becomes more elongated and less flexible when the temperature increases from 20 to 65°C. The excellent fit profiles determined by the EOM modeling process are shown in Fig. 11. The Fig. 12 shows the structure with highest frequency in the optimized population at 20°C superposed to the cloud of models. It was considered the models that covered 80% of the frequency interval.

### Rigid-Body Modeling (RBM)

**Figure 13:**
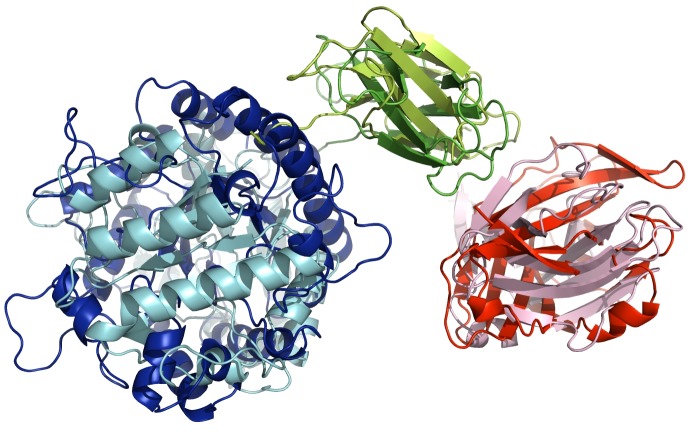
EOM and RBM models. Superposition of the EOM highest frequency model (light blue, light green and pink) and the rigid-body model (RBM) obtained using the CORAL package (dark blue, dark green and red).

The CORAL package [25] was used to perform rigid-body modeling of the three-dimensional structures based on our SAXS curves obtained at different temperatures. The three-dimensional structures of the TpManGH5, TpManIg-like and TpManCBM27 were organized and their relative positions and orientations were optimized by the use of a simulated annealing procedure. As a result, the three-dimensional arrangement of the domains that provides the best fit to SAXS experimental data is obtained. The excellent fits profiles determined by the rigid-body modeling are shown for 20, 50 and 65°C in Fig. 11. The rigid-body model (RBM) obtained at 20°C shows a particle with a *D_max_* of 104.70 Å and *R_g_* = 32.30 Å. The RBM obtained at 50°C shows a particle with a *D_max_* of 111.20 Å and *R_g_* = 33.99 Å. Finally, the RBM obtained at 65°C shows a particle with a *D_max_* of 118.20 Å and *R_g_* = 34.95 Å. Superposition of the low-resolution models obtained at different temperatures by the dummy atom modeling approach (DAM) and the respective TpMan rigid-body models (RBM) show a very good agreement (Fig. 6). Also, the superposition of the EOM highest frequency model and the RBM, both obtained at 20°C and pH 6, shows also a good agreement (Fig. 13).

## Discussion

Modular proteins in which globular domains are connected by linkers are common in nature [26]. It has been recognized that a large number of bacterial and fungal carbohydrases are composed of a catalytic domain and a carbohydrate-binding domain separated by an interdomain linker [27]. A recent study reported that TpMan has a modular structure with a catalytic domain and a carbohydrate-binding domain connected by a long linker (considering its large number of amino acids) [13]. The same study presented the crystal structure of the catalytic domain at 1.5 Å resolution, however, crystallization of the full-length TpMan proved to be unsuccessful, probably owing to the inherent flexibility between domains [13]. In the present study, entire TpMan was analyzed using biophysical techniques as well as with bioinformatics tools. The experiments reported here provided the global structural characterization of the entire TpMan and also the estimation of the influence of the linker and of temperature on the conformation and flexibility of this hyperthermostable endo-β-1,4-mannanase.

### Dimension and Shape of Hyperthermostable Bacterial TpMan at 20°C

The value of the radius of gyration of TpMan (at 20°C and pH 6) calculated from the Guinier plot (*R_g_* = 32±1 Å) is very close to the value obtained from the integral analysis of the scattering curve (*R_g_* = 32.50±0.14 Å) using the method implemented in the GNOM package (Fig. 5). Furthermore, the reconstructed envelope of TpMan restores at 18 Å resolution shows an elongated particle with a *D_max_* of 103.90 Å and *R_g_* = 32.97 Å (Fig. 6). The hydrodynamic radius obtained by DLS (*R_S_* = 36±2 Å) is consistent with the *R_S_* determined for the TpMan low-resolution model (*R_S_* = 38.3 Å) employed the program HYDROPRO [28]. The SAXS data are therefore consistent with a monomeric structure in the form of an elongated L-shaped particle. The values of the radii of gyration and of the *D_max_* of TpMan both indicate that the dimension of the linker is not very large with respect to its mass. Therefore, our results are consistent with a linker with a compact structure and that occupies a small volume with respect to its relative large number of amino acids. Although the resolution of the molecular envelope is limited, it represents the first visualization of a full-length TpMan structure reported to date.

### Molecular Model for the Linker

The low-resolution model obtained by SAXS for the TpMan indicates that the linker has a compact structure. To investigate this hypothesis we performed additional studies employing threading methods and molecular modeling. The templates identified by the servers HHPRED and PHYRE2 are carbohydrases from thermophilic bacteria (Table 3). The central domain (linker) presents low sequence identity when compared with the selected templates (Table 3). However, it is obvious the high level of confidence of the selected templates. TpManCBM27 was directly modeled using as template CBM27 from TmMan [22], which presents high sequence identity and confidence (Table 3). However, a consistent molecular model for TpManCBM27 could be also directly modeled using a template that presents low sequence identity but high confidence, as CBM27 from *Caldicellulosiruptor saccharolyticus*. Similarly, it was possible to obtain a consistent molecular model for the central domain employing X1 domain from CbhA [23] as template (Fig. 8).

The central region (linker) of TpMan is a compact domain of unknown biological function [22]. The molecular model obtained for the central domain is structurally very similar to immunoglobulin-like β-sandwich domain (Ig-like). There is convincing evidence that these structures are simply a “storage form” of the linker that connects other domains of the enzyme that can be extended as needed [23]. Alternatively, they may simply function as spacers between the others domains. In the case of X1 modules from CbhA was proposed that a possible role of these modules is that of thermo structural stabilizers of the CbhA enzyme complex [23]. In the case of TpMan, the deletion of the linker plus TpManCBM27 reduced the thermal stability of the TpManGH5 (Fig. 3), and enzymatic data reveal that the TpManGH5 alone losses significantly more catalytic activity than the full enzyme at elevated temperature (Fig. 1). Thus, a possible role of the central domain is to increase the thermal stability of the whole enzyme as proposed to CbhA complex [23].

### Secondary Structure of Distinct Domains Within Bacterial TpMan

Visual inspection of the CD spectrum of TpManGH5 indicates the presence of α-helical secondary structure as evidenced by negative ellipticities around 210 and 220 nm (Fig. 9B). The spectra are strongly characteristic of an α/β protein, with spectral bands attributed to electron transitions in the amide groups of the protein backbone. Deconvolution of the spectrum using four different algorithms yielded the following average values for the secondary structure: 42±4% α-helix, 16±2 β-sheet, and 42±5% coil (turn plus random). By comparison, the deconvolution results are in a good agreement with published report on the crystal structure of the TpManGH5 (41% α-helix, 12 β-sheet, and 47% coil) [13].

As can be seen from Fig. 9, the CD spectrum for the TpMan does not show two minima well-defined and shows an increase in the negative ellipticities between 210 and 220 nm. These differences are suggestive of a greater β-sheet content for TpMan compared with the TpManGH5, and this difference can only be attributable to the TpManCBM27 plus TpManIg-like. This is reflected by an increase in β-sheet content from 16 to 28% on deconvolution (Table 4). This was readily confirmed by analysis of the molecular models generated for the TpManCBM27 and TpManIg-like (Fig. 8). The TpManIg-like molecular model contains 40% β-sheet and 34% coil, and the TpManCBM27 molecular model contains 6% α-helix, 60% β-sheet, and 34% coil, consistent with the results of secondary-structure prediction (Table 4). Taken together, TpManGH5, TpManIg-like and TpManCBM27 models contain 25% α-helical, 29% β-sheet, and 46% coil, entirely consistent with the results of deconvolution of the experimental spectrum (Table 4).

### Kratky Plot Suggests that TpMan Presents Some Level of Flexibility at 20°C

For a system with a compact shape, the Kratky plot shows a curve with a bell shape, where an initial rising portion is followed by a marked descent thereby forming a well-defined maximum [20]. Nevertheless, the expected curve for a polymer in an extended or random coil conformation rises to a characteristic plateau, with no well-defined maximum [20]. Molecules with compact regions connected by flexible linkers can have both characteristics, a clear maximum followed by plateau regions. The high of the plateau is an indication of the degree of flexibility of the molecule. Thus, one can use the behavior of the Kratky plot to qualitatively assess the degree of flexibility within the scattering molecule [20].

The Kratky plot in Fig. 7A shows that the curve for TpMan, at pH 6 and 20°C, shows a well-defined maximum (*q* <0.15 Å^−1^), however, presents a slight elevated baseline at high *q* values (*q* >0.15Å^−1^). This behavior at *q* >0.15Å^−1^ suggests that TpMan presents some level of intrinsic molecular flexibility. Due to their architecture, modular proteins can often adopt several conformations in solution. The ensemble optimization method (EOM) represents an excellent strategy to identify interdomain motions unambiguously [24]. Thus, to investigate the hypothesis that TpMan presents some level of flexible in solution at 20°C, we performed additional studies employing EOM modeling. The *R_g_* and *D_max_* of the structures selected during the ensemble optimization are centered around 31 Å and 103 Å, respectively (Fig. 10A). The results obtained using EOM (at pH 6 and 20°C) are consistent with a model in which TpMan adopts an ensemble of limited conformations represented by the cloud of models in Fig. 12. The Fig. 13 also shows the structure with highest frequency in the optimized population.

### Three-dimensional Model for the Full-Length TpMan at 20°C

Rigid-body modeling was performed to build a model of the full-length TpMan by fitting of the scattering curve. The three-dimensional structures of the TpManGH5, TpManIg-like and TpManCBM27 were organized and their relative positions and orientations were optimized. The rigid-body adjustments of the three-dimensional structures resulted in an excellent fit to SAXS experimental data (Fig. 11A). Superposition of the low-resolution model obtained by SAXS and TpMan rigid-body model (RBM), both obtained at 20°C and pH 6, shows excellent agreement (Fig. 6A) and it is clear that SAXS model of TpMan is compatible with a monomer composed of three distinct domains. The rigid-body model built shows TpManGH5 and TpManCBM27 occupying the opposite ends of the molecular envelope, while TpManIg-like occupies the central region. Finally, the superposition of the EOM highest frequency model with the rigid-body model obtained using CORAL package indicates that the results are in excellent agreement (Fig. 13). This indicates that, even though the full enzyme has some degree of flexibility at 20°C, there might be a preferable conformation, which could be described by the rigid body modeling procedure.

### SAXS Reveals Temperature-dependent Conformational Changes in TpMan

A better understanding of the biochemistry and biophysics of the processes of conformational change of TpMan as a function of temperature is very important to evaluate the relationship of enzyme structure, stability, flexibility, and enzymatic activity. Little is known about how molecular mobility of carbohydrate-binding domain with respect to catalytic domain correlates with the enzymatic activity. It seems logical to assume that changes in the interplay between the TpManCBM27 and the TpManGH5 can influence the access of polysaccharides to the active site of the enzyme and thus has an impact on the enzymatic activity. In the case of CBM27 from TmMan, it was proposed that the hydrolysis of polysaccharides is enhanced through targeting of the substrate via this carbohydrate-binding domain [22].

The SAXS study presented here demonstrate that TpMan undergoes a temperature-driven transition between conformational states (Fig. 5 and 6) without a significant disruption of its secondary structure (Fig. 9), providing new insight into the function of the enzyme. The increase in average separation between the TpManGH5 and TpManCBM27 as the temperature increase from 20 to 65°C is relatively subtle, but can be seen in the data and models (Fig. 5, 6 and 10). This result indicate that the linker in the case of TpMan can optimize the geometry between the others two domains with respect to the substrate at high temperatures.

## Conclusions

The study reported here establishes the first structural model of a three-domain hyperthermostable bacterial endo-β-1,4-mannanase in solution combining SAXS data analysis and modeling with the knowledge of the three-dimensional atomic resolution structures of each individual domain. Our results shed light on the solution conformation of the entire TpMan enzyme as well as the effect of the length and flexibility of the linker on the spatial arrangement of the constitutive domains. It was demonstrated that the linker occupies a small volume with respect to its relative large number of amino acids. Furthermore, the results also indicate that the linker is a compact domain and structurally very similar to immunoglobulin-like β-sandwich domain. At 20°C, even though the full enzyme has some degree of flexibility, there might be a preferable conformation, which could be described by the rigid body modeling procedure. Furthermore, TpMan undergoes a temperature-driven transition between conformational states without a significant disruption of its secondary structure. Finally, the EOM results indicate that TpMan becomes more elongated and less flexible when the temperature increases from 20 to 65°C. Taken together, the results indicate that the linker in the case of TpMan can optimize the geometry between the others two domains with respect to the substrate at high temperatures.

Based on these observations we advocate that the linker is very important to guarantee the optimal conditions for TpMan enzymatic activity due to at least three factors: *i*) the thermostabilization of the whole enzyme, *ii*) adequate degree of molecular flexibility between the structural domains and *iii*) optimize the geometry between the others domains with respect to the substrate at high temperatures. These studies should provide a useful basis for future biophysical studies of entire TpMan and others multimodular carbohydrases.

## Materials and Methods

### Materials

Nickel-nitrilotriacetic acid resin (Ni-NTA), imidazole, kanamycin, LB medium, isopropyl-β-D-thiogalactopyranoside (IPTG) and locust bean gum were purchased from Sigma-Aldrich. All chemicals and reagents used in this study were of the highest purity analytical grade.

### Expression and Purification of Recombinant TpMan and TpManGH5

The expression and purification of endo-β-1,4-mannanase (TpMan) and its isolated catalytic core domain (TpManGH5) were carried out as described previously with minor modification [13], and the purity of the final product verified by SDS-PAGE. The resulting TpMan and TpManGH5 were exhaustively dialyzed in 20 mM acetate-borate-phosphate buffer adjusted at pH 6 for elimination of imidazole and NaCl. The concentrations of the recombinant proteins were determined by UV absorbance at λ = 280 nm using a theoretical extinction coefficient based on the amino acid composition. The theoretical coefficients employed were ε_280 nm_ = 156,900 M^−1^cm^−1^ for TpMan and ε_280 nm_ = 108,875 M^−1^cm^−1^ for TpManGH5. The final products TpMan and TpManGH5 were then frozen and stored at −80°C and were melted on ice before use.

### Enzymatic Activity Measurements

Mannan endo-1,4-β-mannosidase activity was determined using as substrate 1% against locust bean gum (galactomannan) dissolved in a buffer. Activity of TpMan and TpManGH5 were measured in the temperature range between 20 and 85°C (20 mM acetate-borate-phosphate buffer adjusted at pH 6). The reaction in each experiment was performed by mixing 20 μL of the diluted enzyme (1.5 μM) with 80 μL of galactomannan for 10 min. The reaction was stopped by the addition of DNS reagent followed by boiling the samples at 100°C water bath for 5 min [29]. One unit of mannan endo-1,4-β-mannosidase activity was defined as the amount of enzyme needed to release 1 μmol of mannose equivalent per minute. All experiments were done in triplicate, and average values are reported.

### Dynamic Light Scattering (DLS)

The size characteristic of the purified TpMan and TpManGH5 samples were examined by means of the Nano-ZS dynamic light scattering system (Malvern Instruments Ltd, Malvern, UK). This system employs a 633 nm laser and a fixed scattering angle (173°). Protein solutions (between 0.5 and 8 mg/mL), in 20 mM acetate-borate-phosphate buffer adjusted at pH 6, were first passed through a 0.22 μm filter (Millipore, USA), centrifuged at 16,000×g for 10 min at room temperature, and subsequently loaded into a cuvette prior to measurement. The temperature was raised from 20 to 85°C and the samples were allowed to equilibrate for 2 min in each temperature prior to DLS measurements, after which multiple records of the DLS profile were collected. In each case the hydrodynamic radius was obtained from a second order cumulant fit to the intensity auto-correlation function (size distribution by volume).

### Small-Angle X-ray Scattering (SAXS) Data Collection

For SAXS measurements, TpMan was measured at different protein concentrations (2, 4 and 8 mg/mL in 20 mM acetate-borate-phosphate buffer adjusted at pH 6) and temperatures (T = 20, 50 and 65°C). The samples were passed through a 0.22 μm filter (Millipore, USA) and centrifuged at 16,000×g for 10 min at room temperature prior to measurement. No concentration effects were detected for the samples. The measurements were carried out on a laboratory SAXS instrument Bruker-Nanostar, placed at the Institute of Physics of University of São Paulo. This equipment is improved by the use of microfocus source Genix3D coupled with Fox3D multilayer optics and two sets of scatter less slits for beam definition, all provided by Xenocs. The samples were kept on a quartz capillary glued to a stainless steel case, which enabled the proper rinse and reuse of the sample holder, permitting an accurate background subtraction. The scattering of water measured on the same sample holders was used to normalize the data to absolute scale. The sample temperature was controlled by a Peltier system. Several 900 s frames were recorded for each sample to monitor radiation damage and beam stability. The wavelength of the incoming monochromatic X-ray beam was λ = 1.54 Å (Cu_kα_) and the sample to detector distance was 0.67 m, providing an *q* (scattering vector) interval from 0.008 to 0.35 Å^−1^, where *q* = 4 π sin(θ)/λ and θ is half the scattering angle. The 2D scattering data was collected on a Vantec2000 detector and the integration of the SAXS patterns were performed by the use of the Bruker SAXS software. The data treatment, normalization to absolute scale and averaging procedures were performed by the use of the SUPERSAXS package [Oliveira and Pedersen, unpublished].

### SAXS Data Analysis

The radius of gyration (*R_g_*) of the molecules were determined by two independent procedures: *i*) by the Guinier equation *I*(*q*) = *I*(0).exp[(−*q* ^2^.*R_g_* ^2^)/3], *q*<1.3/*R_g_*, and *ii*) by the indirect Fourier transform method using the GNOM package (www.embl-hamburg.de/biosaxs) [17]. The distance distribution function *P*(r) was also evaluated with GNOM software and the maximum diameter (*D_max_*) was obtained.

### SAXS *ab initio* Modeling

Dummy atom models (DAMs) were calculated from the experimental SAXS by *ab initio* procedures implemented in DAMMIN package (www.embl-hamburg.de/biosaxs) [18]. The low-resolution models obtained, in each case, were compared with each other by the use of the DAMAVER [19] procedure and the most representative model for the whole set was used. The resolution (R) was estimated from the equation R = 2π/*q_max_*. The CRYSOL package (www.embl-hamburg.de/biosaxs) was used to generate theoretical scattering curves from DAMs [30]. *R_g_* and *D_max_* were determined with the same package.

### Rigid-Body Modeling and EOM Modeling

Rigid-body modeling of the TpMan was performed with the CORAL package [25]. CRYSOL was used to generate the simulated scattering curves from the rigid-body model. The rigid-body model and *ab initio* DAMs were superimposed with de SUPCOMB package [31]. Additional structural modeling employed the ensemble optimization modeling method (EOM) described by Bernadó *et al.* [24]. Superposition figures were generated by the PyMOL program (www.pymol.org).

### Molecular Modeling

The remote homology detection servers HHPRED [32] and PHYRE2 [33] were used to search for homologs of the last 274 amino acids residues from TpMan in the Protein Data Bank (PDB) with default parameters. Molecular models for TpManCBM27 and TpManIg-like were built using restraint-based homology modeling, as implemented in the program Modeller software [34]. The alignment of the TpManCBM27 against the sequence of CBM27 from *Thermotoga maritima* endo-β-1,4-mannanase was initially used as input to the Modeller program, together with the atomic coordinates of the latter (PDB 1OH4). The alignment of the TpManIg-like against the sequence of X1 domain from *Clostridium thermocellum* cellobiohydrolase A was initially used as input to the Modeller program, together with the atomic coordinates of the latter (PDB 3PE9).

### Computational Methods for Secondary-structure Prediction

The theoretical extinction coefficients, based on the amino acid composition (Gene Bank: ABQ47550.1), for TpMan and TpManGH5 were obtained from the ProtParam utility available on the ExPaSy server (www.expasy.org). The methods used for general secondary-structure prediction were PHD [35], PSIPRED [36], BALDIG [37], and PORTER [38].

### Far-UV Circular Dichroism (CD) Spectroscopy

Far-UV CD spectra were collected using a JascoJ-815 spectropolarimeter equipped with a temperature control device. TpMan and TpManGH5 concentrations were 5 μM in 20 mM acetate-borate-phosphate buffer adjusted at pH 6. All data were collected using 0.1 cm quartz cuvette and the spectra were recorded over the wavelength range from 195 to 250 nm. In the case of TpMan, CD measurements were collected at different temperature values (T = 20, 65 and 85°C). Eight accumulations were averaged to form the CD spectra, taken using a scanning speed of 100 nm min^−1^, a spectral bandwidth of 1 nm, and a response time of 0.5 s, and obtained on degree scale. The buffer contribution was subtracted in each of the experiments. Spectra were transformed to molar ellipticity ^hello^θ] using the mean weight residue and concentration prior to the secondary-structure analysis. To obtain structural information, CD spectra were deconvoluted using the SELCON3 [39], CONTIN [40], CDS [41], and K2D3 [42] programs using different databases.

## Supporting Information

Figure S1 **DLS temperature experiments with TpMan.** (**A**) The size distribution by intensity for purified TpMan (0.5 mg/mL) where DLS runs were conducted between 20–85°C. (**B**) The size distribution by volume for purified TpMan where DLS runs were conducted at 20, 30, 40, 50 and 85°C.
(TIF)

Figure S2 **DLS temperature experiments with TpManGH5.** (**A**) The size distribution by intensity for purified TpManGH5 (0.5 mg/mL) where DLS runs were conducted between 20–70°C. (**B**) The size distribution by volume for purified TpManGH5 where DLS runs were conducted at 20, 30, 40, 50 and 70°C.
(TIF)

## References

[1] Chang MC (2007) Harnessing energy from plant biomass. Curr. Opin. Chem. Biol. 11: 677–684. 10.1016/j.cbpa.2007.08.039 17942363

[2] Rezende CA, de Lima MA, Maziero P, Deazevedo ER, Garcia W, et al (2011) Chemical and morphological characterization of sugarcane bagasse submitted to a delignification process for enhanced enzymatic digestibility. Biotechnol. Biofuels 4 54: 1–18. 10.1186/1754-6834-4-54 PMC3377919 22122978

[3] Lima MA, Lavorente GB, da Silva HK, Bragatto J, Rezende CA, et al (2013) Effects of pretreatment on morphology, chemical composition and enzymatic digestibility of eucalyptus bark: a potentially valuable source of fermentable sugars for biofuel production - part 1. Biotechnol. Biofuels 6 75: 1–17. 10.1186/1754-6834-6-75 PMC3667114 23657132

[4] Saha BC (2003) Hemicellulose bioconversion. J. Ind. Microbiol. Biotechnol. 30: 279–291. 10.1007/s10295-003-0049-x 12698321

[5] Polizeli MLT, Rizzati ACS, Monti R, Terenzi HF, Jorge JA, et al (2005) Xylanases from fungi: properties and industrial applications. Appl. Microbiol. Biotechnol. 67: 577–591. 10.1007/s00253-005-1904-7 15944805

[6] Gírio FM, Fonseca C, Carvalheiro F, Duarte LC, Marques S, et al (2010) Hemicelluloses for fuel ethanol: a review. Bioresource Technol. 101: 4775–4800. 10.1016/j.biortech.2010.01.088 20171088

[7] Moreira LRS, Filho EXF (2008) An overview of mannan structure and mannan-degrading enzyme systems. Appl. Microbiol. Biotechnol. 79: 165–178. 10.1007/s00253-008-1423-4 18385995

[8] van Zyl WH, Rose SH, Trollope K, Görgens JF (2010) Fungal β-mannanases: mannan hydrolysis, heterologous production and biotechnological applications. Process Biochemistry 45: 1203–1213.

[9] Tenkanen M, Makkonen M, Perttula M, Viikari L, Teleman A (1997) Action of *Trichoderma ressei* mannanase on galactoglucomannan in pine kraft pulp. J. Biotechnol. 57: 191–204. 10.1016/s0168-1656(97)00099-0 9335173

[10] Montiel MD, Hernández M, Rodríguez J, Arias ME (2002) Evaluation of an endo-beta-mannase produced by *Streptomyces ipomoea* CECT for the biobleaching of pine kraft pulps. Appl. Microbiol. Biotechnol. 58: 67–72. 10.1007/s00253-001-0866-7 11831476

[11] Dhawan S, Kaur J (2007) Microbial mannanases: an overview of production and applications. Crit. Rev. Biotechnol. 27: 197–216. 10.1080/07388550701775919 18085462

[12] Takahata Y, Nishijima M, Hoaki T, Maruyama T (2001) *Thermotoga petrophila* sp. nov. and *Thermotoga naphthophila* sp. nov., two hyperthermophilic bacteria from the Kubiki oil reservoir in Niigata, Japan. Int. J. Syst. Evol. Microbiol. 51: 1901–1909. 10.1099/00207713-51-5-1901 11594624

[13] Santos CR, Paiva JH, Meza AN, Cota J, Alvarez TM, et al (2012) Molecular insights into substrate specificity and thermal stability of a bacterial GH5-CBM27 endo-1,4-β-D-mannanase. J. Struct. Biol. 177: 469–476. 10.1016/j.jsb.2011.11.021 22155669

[14] Cota J, Alvarez TM, Citadini AP, Santos CR, Oliveira-Neto M, et al (2011) Mode of operation and low resolution structure of a multi-domain and hyperthermophilic endo-β-1,3-glucanase from *Thermotoga petrophila*. Biochem. Biophys. Res. Commun. 406: 590–594. 10.1016/j.bbrc.2011.02.098 21352806

[15] Santos CR, Squina FM, Navarro AM, Oldiges DP, Leme AF, et al (2011) Functional and biophysical characterization of hyperthermostable GH5 a-L-arabinofuranosidase from *Thermotoga petrophila*. Biotechnol. Lett. 33: 131–137. 10.1007/s10529-010-0409-3 20872163

[16] Hall M, Rubin J, Behrens SH, Bommarius AS (2011) The cellulose-binding domain of cellobiohydrolase Cel7A from *Trichoderma reesei* is also a thermostabilizing domain, J. Biotechnol. 155: 370–376. 10.1016/j.jbiotec.2011.07.016 21807036

[17] Svergun DI (1992) Determination of the regularization parameter inindirect-transform methods using perceptual criteria. J. Appl. Cryst. 25: 495–503.

[18] Svergun DI (1999) Restoring low resolution structure of biological macromolecules from solution scattering using simulated annealing. Biophys. J. 76: 2879–2886. 10.1016/S0006-3495(99)77443-6 PMC1300260 10354416

[19] Volkov VV, Svergun DI (2003) Uniqueness of *ab-initio* shape determination in small-angle scattering. J. Appl. Cryst. 36: 860–864. 10.1107/S0021889809000338 PMC5023043 27630371

[20] Rambo RP, Tainer JA (2011) Characterizing flexible and intrinsically unstructured biological macromolecules by SAS using the Porod-Debye law. Biopolymers 95: 559–571. 21509745 10.1002/bip.21638 PMC3103662

[21] Bernadó P (2010) Effect of interdomain dynamics on the structure determination of modular proteins by small-angle scattering. Eur. Biophys. J. 39: 769–780. 10.1007/s00249-009-0549-3 19844700

[22] Boraston AB, Revett TJ, Boraston CM, Nurizzo D, Davies GJ (2003) Structural and thermodynamic dissection of specific mannan recognition by a carbohydrate binding module, TmCBM27. Structure 11: 665–675. 12791255 10.1016/s0969-2126(03)00100-x

[23] Brunecky R, Alahuhta M, Bomble YJ, Xu Q, Baker JO, et al (2012) Structure and function of the *Clostridium thermocellum* cellobiohydrolase A X1-module repeat: enhancement through stabilization of the CbhA complex, Acta Crystallogr. D Biol. Crystallogr. 68: 292–299. 10.1107/S0907444912001680 22349231

[24] Bernado P, Mylonas E, Petoukhov MV, Blackledge M, Svergun DI (2007) Structural Characterization of Flexible Proteins Using Small-Angle X-ray Scattering. J. Am. Chem. Soc. 129: 5656–5664. 10.1021/ja069124n 17411046

[25] Petoukhov MV, Franke D, Shkumatov AV, Tria G, Kikhney AG, et al (2012) New developments in the ATSAS program package for small-angle scattering data analysis. J. Appl. Cryst. 45: 342–350. 10.1107/S0021889812007662 PMC4233345 25484842

[26] Levitt M (2009) Nature of the protein universe. Proc. Natl. Acad. Sci. USA 106: 11079–11084. 10.1073/pnas.0905029106 PMC2698892 19541617

[27] Gilkes NR, Henrissat B, Kilburn DG, Warren RA (1991) Domains in microbial β-1,4-glycanases: sequence conservation, function, and enzyme families. Microbiol. Rev. 55: 303–315. 10.1128/mr.55.2.303-315.1991 PMC372816 1886523

[28] De La Torre JG, Huertas KL, Carrasco B (2000) Calculation of hydrodynamic properties of globular proteins from their atomic-level structure. Biophys J. 78: 719–730. 10.1016/S0006-3495(00)76630-6 PMC1300675 10653785

[29] Miller GL (1959) Use of dinitrosalicylic acid reagent for determination of reducing sugar. Anal. Chem. 31: 426–428.

[30] Svergun DI, Barberato C, Koch MHJ (1995) CRYSOL - a Program to Evaluate X-ray Solution Scattering of Biological Macromolecules from Atomic Coordinates. J. Appl. Cryst. 28: 768–773.

[31] Kozin MB, Svergun DI (2001) Automated matching of high- and low-resolution structural models. J. Appl. Crystallogr. 34: 33–41.

[32] Söding J, Biegert A, Lupas AN (2005) The HHpred interactive server for protein homology detection and structure prediction. Nucleic Acids Res. 33: W244–248. 10.1093/nar/gki408 PMC1160169 15980461

[33] Kelley LA, Sternberg MJ (2009) Protein structure prediction on the Web: a case study using the Phyre server. Nat. Protoc. 4: 363–371. 10.1038/nprot.2009.2 19247286

[34] Sali A, Blundell TL (1993) Comparative protein modelling by satisfaction of spatial restraints. J. Mol. Biol. 234: 779–815. 10.1006/jmbi.1993.1626 8254673

[35] Rost B (1996) PHD-Predicting one-dimensional protein structure by profile-based neural networks. Methods Enzymol. 266: 525–539. 10.1016/s0076-6879(96)66033-9 8743704

[36] Jones DT (1999) PSIPRED-Protein secondary structure prediction based on position-specific scoring matrices. J. Mol. Biol. 292: 195–202. 10.1006/jmbi.1999.3091 10493868

[37] Randall A, Baldi P (2008) SELECTpro: effective protein model selection using a structure-based energy function resistant to BLUNDERs. BMC Struc. Biol. 8: 1–16. 10.1186/1472-6807-8-52 PMC2667183 19055744

[38] Pollastri G, McLysaght A (2005) Porter - a new, accurate server for protein secondary structure prediction. Bioinformatics 21: 1719–1720. 15585524 10.1093/bioinformatics/bti203

[39] Sreerama N, Woody RW (1993) A self-consistent method for the analysis of protein secondary structure from circular dichroism. Anal. Biochem. 209: 32–44. 10.1006/abio.1993.1079 8465960

[40] Provencher SW, Glockner J (1981) Estimation of globular protein secondary structure from circular dichroism. Biochemistry 20: 33–37. 7470476 10.1021/bi00504a006

[41] Johnson WC (1999) Analyzing protein circular dichroism spectra for accurate secondary structures. Proteins: Struct. Funct. Genet. 35: 307–312. 10328265

[42] Perez-Iratxeta C, Andrade-Navarro MA (2008) K2D2 - estimation of protein secondary structure from circular dichroism spectra. BMC Struct. Biol. 8: 1–5. 10.1186/1472-6807-8-25 PMC2397409 18477405

