# Supplementary figures and images for: Modular Hyperthermostable Bacterial Endo-β-1,4-Mannanase: Molecular Shape, Flexibility and Temperature-Dependent Conformational Changes
Source: PLoS One. 2014 Mar 26;9(3):e92996. doi: 10.1371/journal.pone.0092996 (PMC3966859; doi:10.1371/journal.pone.0092996)

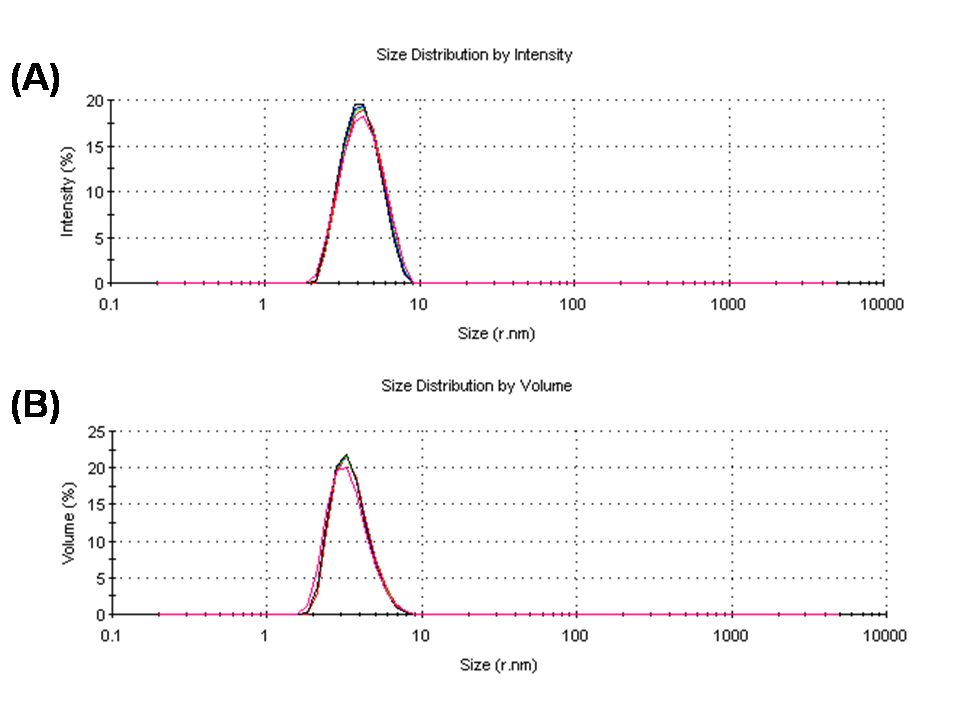

Supplement: Figure S1 — DLS temperature experiments with TpMan. (A) The size distribution by intensity for purified TpMan (0.5 mg/mL) where DLS runs were conducted between 20–85°C. (B) The size distribution by volume for purified TpMan where DLS runs were conducted at 20, 30, 40, 50 and 85°C. (TIF) [file pone.0092996.s001.tif]

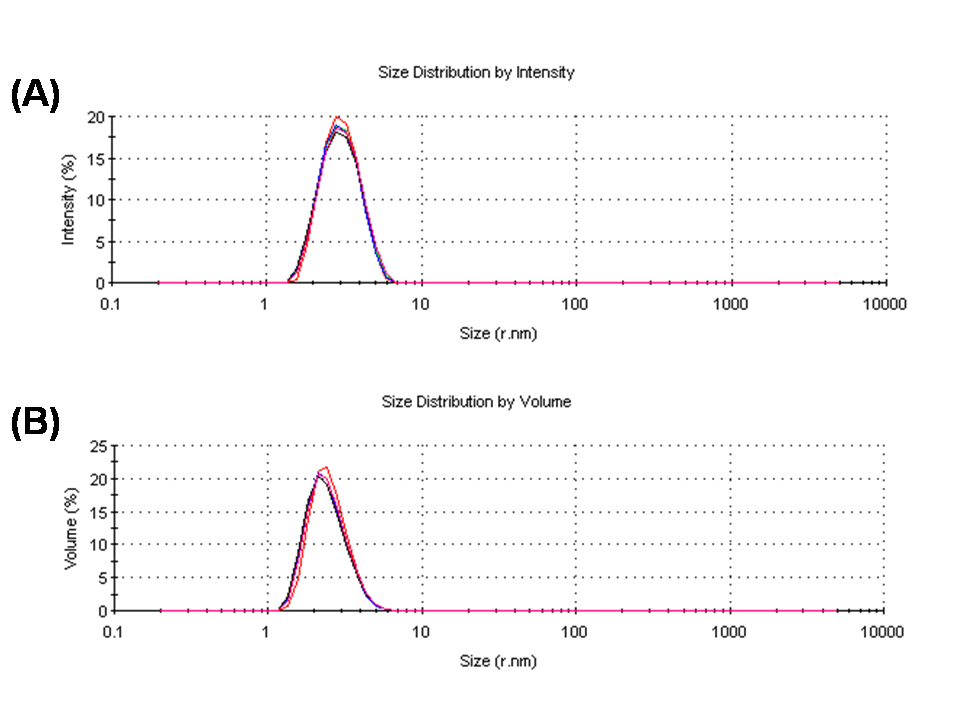

Supplement: Figure S2 — DLS temperature experiments with TpManGH5. (A) The size distribution by intensity for purified TpManGH5 (0.5 mg/mL) where DLS runs were conducted between 20–70°C. (B) The size distribution by volume for purified TpManGH5 where DLS runs were conducted at 20, 30, 40, 50 and 70°C. (TIF) [file pone.0092996.s002.tif]
